# Cell Type-Specific Expression of Corticotropin-Releasing Hormone-Binding Protein in GABAergic Interneurons in the Prefrontal Cortex

**DOI:** 10.3389/fnana.2017.00090

**Published:** 2017-10-10

**Authors:** Kyle D. Ketchesin, Nicholas S. Huang, Audrey F. Seasholtz

**Affiliations:** ^1^Neuroscience Graduate Program, University of Michigan, Ann Arbor, MI, United States; ^2^Molecular and Behavioral Neuroscience Institute, University of Michigan, Ann Arbor, MI, United States; ^3^Department of Biological Chemistry, University of Michigan, Ann Arbor, MI, United States

**Keywords:** corticotropin releasing hormone, corticotropin releasing hormone-binding protein, CRH-BP, interneurons, somatostatin, prefrontal cortex

## Abstract

Corticotropin-releasing hormone-binding protein (CRH-BP) is a secreted glycoprotein that binds CRH with very high affinity to modulate CRH receptor activity. CRH-BP is widely expressed throughout the brain, with particularly high expression in regions such as the amygdala, hippocampus, ventral tegmental area and prefrontal cortex (PFC). Recent studies suggest a role for CRH-BP in stress-related psychiatric disorders and addiction, with the PFC being a potential site of interest. However, the molecular phenotype of CRH-BP-expressing cells in this region has not been well-characterized. In the current study, we sought to determine the cell type-specific expression of CRH-BP in the PFC to begin to define the neural circuits in which this key regulator is acting. To characterize the expression of CRH-BP in excitatory and/or inhibitory neurons, we utilized dual *in situ* hybridization to examine the cellular colocalization of CRH-BP mRNA with vesicular glutamate transporter (VGLUT) or glutamic acid decarboxylase (GAD) mRNA in different subregions of the PFC. We show that CRH-BP is expressed predominantly in GABAergic interneurons of the PFC, as revealed by the high degree of colocalization (>85%) between CRH-BP and GAD. To further characterize the expression of CRH-BP in this heterogenous group of inhibitory neurons, we examined the colocalization of CRH-BP with various molecular markers of GABAergic interneurons, including parvalbumin (PV), somatostatin (SST), vasoactive intestinal peptide (VIP) and cholecystokinin (CCK). We demonstrate that CRH-BP is colocalized predominantly with SST in the PFC, with lower levels of colocalization in PV- and CCK-expressing neurons. Our results provide a more comprehensive characterization of the cell type-specific expression of CRH-BP and begin to define its potential role within circuits of the PFC. These results will serve as the basis for future *in vivo* studies to manipulate CRH-BP in a cell type-specific manner to better understand its role in stress-related psychiatric disorders, including anxiety, depression and addiction.

## Introduction

Corticotropin-releasing hormone (CRH) is the key central nervous system regulator of the mammalian stress response. CRH mediates its effects through binding to two G-protein-coupled receptors, CRH receptor 1 (CRH-R1) and CRH receptor 2 (CRH-R2). The activity of CRH is also modulated by CRH-binding protein (CRH-BP), a 37-kDa secreted glycoprotein that is structurally distinct from the CRH receptors. This evolutionarily conserved protein binds CRH and the CRH-like ligand urocortin 1 with a greater affinity than the CRH receptors. Multiple roles have been proposed for the CRH-BP (reviewed in Westphal and Seasholtz, 2006; Ketchesin et al., 2017). In cultured pituitary cells, CRH-BP attenuates CRH-R1-mediated ACTH release, demonstrating an inhibitory role for CRH-BP at CRH-R1 (Potter et al., 1991; Cortright et al., 1995; Sutton et al., 1995). Other studies have suggested a potential facilitatory role for CRH-BP at CRH-R2, particularly in the ventral tegmental area (VTA; Ungless et al., 2003; Wang et al., 2007; Albrechet-Souza et al., 2015). Additional studies suggest that CRH-BP may have actions independent of CRH receptor (Chan et al., 2000) or may act as an escort protein to traffic CRH-R2α to the cell surface (Slater et al., 2016). Thus, the role of CRH-BP and its mechanism of action may depend on a variety of factors, including CRH receptor subtype, brain region, or specific cell type.

CRH-BP is widely expressed throughout the brain, including the cerebral cortex, amygdala, hippocampus, VTA, and a variety of brainstem nuclei (Potter et al., 1992; Chan et al., 2000). CRH-BP is expressed in several regions where CRH is expressed, such as the bed nucleus of the stria terminalis (BNST) and the central nucleus of the amygdala, suggesting potential sites of interaction (Potter et al., 1992). Moreover, CRH-BP is expressed in a number of CRH target sites where the CRH receptors are expressed, including the anterior pituitary, basolateral amygdala, VTA and medial prefrontal cortex (mPFC; Potter et al., 1992; Stinnett et al., 2015; Ketchesin et al., 2016). Both rodent and human studies have implicated a role for CRH-BP in stress-related psychiatric disorders, including anxiety, depression and addiction (Enoch et al., 2008; Binder et al., 2010; Albrechet-Souza et al., 2015; Haass-Koffler et al., 2016; Ketchesin et al., 2016, 2017). We have recently shown that repeated cycles of binge drinking in mice decrease CRH-BP mRNA expression in the mPFC (Ketchesin et al., 2016). This structure is involved in executive function and regulation of emotion and behavior, including responses to stress, with the prelimbic and infralimbic subregions often exhibiting opposing roles (Radley et al., 2006; Jaferi and Bhatnagar, 2007; McKlveen et al., 2015). Other studies have shown that the CRH system in the mPFC plays an important role in stress-related behaviors, including anxiety, learning and memory and excessive alcohol consumption (Jaferi and Bhatnagar, 2007; George et al., 2012; Glaser et al., 2014; Gondré-Lewis et al., 2016; Uribe-Mariño et al., 2016). While CRH and CRH-R1 have been localized to specific cell types in the PFC, the molecular phenotype of CRH-BP-expressing cells in this region has not been well-characterized.

The PFC contains two broad classes of neurons: glutamatergic excitatory pyramidal neurons and GABAergic inhibitory interneurons. Although GABAergic interneurons represent only 10%–20% of neurons in the cortex, they play a critical role in modulating the output of the cortical excitatory pyramidal neurons (Xu et al., 2010). Interneurons are highly diverse and can be subdivided into different classes based on properties such as morphology, electrophysiology, connectivity and neuropeptide/calcium-BP marker expression (Ascoli et al., 2008). Molecular markers commonly used to identify interneurons include the calcium-BP parvalbumin (PV) and the neuropeptides somatostatin (SST), vasoactive intestinal peptide (VIP) and cholecystokinin (CCK), which account for the majority of interneurons in the frontal cortex (Kawaguchi and Kubota, 1997). SST, PV and VIP compose three distinct non-overlapping classes of interneurons, while there is partial overlap between CCK and VIP interneurons (Kawaguchi and Kubota, 1997; Xu et al., 2010). CRH is expressed in SST-, VIP- and VIP/CCK-expressing interneurons in the cortex, while CRH-R1 is localized in glutamatergic pyramidal neurons (Gallopin et al., 2006; Kubota et al., 2011; Refojo et al., 2011; Mardinly et al., 2016). The PFC also receives CRH afferents from other brain regions, including the laterodorsal tegmental nucleus (Crawley et al., 1985), demonstrating multiple sources for CRH in this region. Together, these expression patterns suggest an important role for the CRH system in PFC circuitry.

The goal of the current study was to determine the cell type-specific expression of CRH-BP in the PFC to begin to define the neural circuits in which CRH-BP is acting. We utilized dual *in situ* hybridization to determine the expression of CRH-BP mRNA in excitatory (vesicular glutamate transporter (VGLUT)-positive) or inhibitory (glutamic acid decarboxylase (GAD)-positive) neurons in different regions of the PFC to determine whether CRH-BP is acting locally within the PFC or projecting to other brain regions to mediate its effects. We find that CRH-BP mRNA colocalizes predominantly with GAD, revealing the presence of CRH-BP in inhibitory interneurons of the PFC. To further characterize the expression of CRH-BP in inhibitory neurons of the PFC, we examined the colocalization of CRH-BP with the interneuron molecular markers PV, SST, CCK, and VIP and show that CRH-BP colocalizes predominantly with SST, with lower levels of coexpression in PV and CCK neurons.

## Materials and Methods

### Animals

Male C57BL/6J mice (The Jackson Laboratory, Bar Harbor, ME, USA) were used for all experiments. Mice (*n* = 5/experiment) were 10- to 20-weeks old at the time of experiments. Mice were maintained on a 14-h light/10-h dark cycle and had access to food and water *ad libitum*. All mouse experiments were conducted according to National Institutes of Health guidelines for animal care and were approved by the University of Michigan Committee on Use and Care of Animals.

### *In Situ* Hybridization Riboprobes

A VGLUT1 antisense cRNA riboprobe was generated from a pCRII-TOPO plasmid containing a 745-bp fragment of mouse VGLUT1 cDNA (mVGLUT1-pTOPO; GenBank accession no. NM_182993, nucleotides 209–953). A VGLUT2 antisense cRNA riboprobe was synthesized from a pCRII-TOPO plasmid containing a 840-bp fragment of mouse VGLUT2 cDNA (mVGLUT2-pTOPO; accession no. NM_080853, nucleotides 901–1741). For antisense riboprobe synthesis, the mVGLUT1-pTOPO and mVGLUT2-pTOPO plasmids were linearized with HindIII and BamHI, respectively, and transcribed with T7 RNA polymerase (Promega, Madison, WI, USA). A GAD65 antisense cRNA riboprobe was generated from a BSSK plasmid containing a 613-bp fragment of mouse GAD65 cDNA (mGAD65-BSSK; accession no. NM_008078, nucleotides 1221–1834). A GAD67 antisense cRNA riboprobe was synthesized from a BSSK plasmid containing a 651-bp fragment of mouse GAD67 cDNA (mGAD67-BSSK; accession no. NM_008077, nucleotides 2354–3005). mGAD65-BSSK and mGAD67-BSSK plasmids were kindly provided by Dr. Stanley Watson, University of Michigan, Ann Arbor, MI, USA. For antisense riboprobe synthesis, the mGAD65-BSSK and mGAD67-BSSK plasmids were linearized with BamHI and transcribed with T7 RNA polymerase. VGLUT1, VGLUT2, GAD65 and GAD67 riboprobes were generated with the following transcription reaction: 6 μl 5× transcription buffer, 2 μl 100 mM DTT, 1 μl each 10 mM ATP, CTP and GTP, 1 μl 10 mM digoxigenin-11-UTP (4:6 digoxigenin-11-UTP:UTP ratio; Roche Diagnostics, Indianapolis, IN, USA), 1.5 μl linearized DNA (1 μg/μl), 1 μl RNA polymerase (20 units/μl), and 1 μl RNase inhibitor (RNaseOUT; 40 units/μl) in a 30 μl reaction volume. All riboprobes were purified using Micro Bio-Spin P-30 columns (Bio-Rad Laboratories, Hercules, CA, USA) and recovered in a 40 μl volume of water.

PV, VIP and CCK cDNA sequences were isolated from mouse cortex cDNA by PCR using Taq polymerase. The primers used to isolate PV, VIP and CCK cDNA were as follows: PV—5′-TTTGCTGCTGCAGACTCCTT-3′ and 5′-TCTACTATACCCCCACTGCCC-3′ (555-bp product, accession no. NM_013645.3, nucleotides 77–631); VIP—5′-TTGGCAAACGAATCAGCAGC-3′ and 5′-TCCTCGATTGCTACCCTTGC-3′ (531 bp-product, accession no. NM_011702.2, nucleotides 486–1016); CCK—5′-GGTGATGGCAGTCCTAGCTG-3′ and 5′-AAGGAAACACTGCCTTCCGA-3′ (508 bp-product, accession no. NM_031161.4, nucleotides 126–633). The PCR products were subcloned into the pCR-II TOPO vector (TOPO-TA kit; 45-0640, Invitrogen, Carlsbad, CA, USA) and confirmed by restriction digests and DNA sequencing. For riboprobe synthesis, the mCCK-pTOPO and mVIP-pTOPO plasmids were linearized with BamHI and transcribed with T7 RNA polymerase. The mPV-pTOPO plasmid was linearized with XhoI and transcribed with SP6 RNA polymerase (Promega, Madison, WI, USA). A SST antisense cRNA riboprobe was generated from a BSSK plasmid containing a 390-bp fragment of rat SST cDNA (rSST; accession no. NM_012659, nucleotides 110–500; kindly provided by Dr. Stanley Watson, University of Michigan, Ann Arbor, MI, USA). The rSST plasmid was linearized with BamHI and transcribed with T7 RNA polymerase. SST, PV, CCK and VIP riboprobes were labeled with digoxigenin-11-UTP (4:6 digoxigenin-11-UTP:UTP ratio) as described above. A CRH-BP antisense cRNA riboprobe was synthesized from a pGEM-3Z plasmid containing a 666-bp fragment of mouse CRH-BP cDNA (mCRHBP666; accession no. NM_198408, nucleotides 372–1037; Cortright et al., 1995). The mCRHBP666 plasmid was linearized with XhoI and transcribed with T7 RNA polymerase. The CRH-BP riboprobe was generated with the following transcription reaction: 6.4 μl ^35^S-UTP (1250 Ci/mmol; PerkinElmer Inc., Waltham, MA, USA), 6 μl 5× transcription buffer, 2 μl 100 mM DTT, 1 μl each 10 mM CTP, ATP and GTP, 1.5 μl linearized DNA (1 μg/μl), 1 μl T7 RNA polymerase (20 units/μl) and 1 μl RNase inhibitor (RNaseOUT; 40 units/μl) in a 30 μl reaction volume. All riboprobes were purified using Micro Bio-Spin P-30 columns (Bio-Rad Laboratories, Hercules, CA, USA) and recovered in a 40 μl volume of water.

### Tissue Processing and Dual *in Situ* Hybridization

Brains from male mice were sectioned at 14 μm and collected in a series of six slides (four sections/slide). Every sixth slide was stained with cresyl violet to determine anatomical location. The cell type-specific expression of CRH-BP in the PFC was determined using dual *in situ* hybridization, similar to what has been described previously (Speert et al., 2002). Slides were postfixed in 4% paraformaldehyde for 1 h, followed by three washes in 2× saline sodium citrate (SSC) buffer. Slides were then incubated in 0.25% acetic anhydride in 0.1 M triethanolamine (pH 8) for 10 min, washed three times in 2× SSC, dehydrated in ethanol, and then air-dried. To determine the cellular colocalization of CRH-BP with VGLUT or GAD (Experiment 1), adjacent slides containing PFC were hybridized with the ^35^S-labeled CRH-BP riboprobe (2 × 10^6^ cpm/slide) and digoxigenin-labeled riboprobes in 50% formamide hybridization buffer (Ameresco, Framingham, MA, USA) with 20 mM DTT at 55°C overnight. For the VGLUT hybridization reaction, the ^35^S-labeled CRH-BP riboprobe (2 × 10^6^ cpm/slide) was combined with the digoxigenin-labeled VGLUT1 (1.5 μl/slide) and VGLUT2 (1.5 μl/slide) riboprobes. For the GAD hybridization reaction, the ^35^S-CRH-BP riboprobe was combined with the digoxigenin-labeled GAD65 and GAD67 riboprobes (1.5 μl each/slide). To determine the cellular colocalization of CRH-BP with various interneuron molecular markers (Experiment 2), adjacent slides were hybridized with the ^35^S-labeled CRH-BP riboprobe (2 × 10^6^ cpm/slide) and a single digoxigenin-labeled riboprobe (CCK, VIP, PV, or SST; 1.5 μl/slide) in 50% formamide hybridization buffer with 20 mM DTT at 55°C overnight. After hybridization, excess unhybridized probe was removed by three 2× SSC washes and sections were incubated in RNase A (200 μg/ml; Sigma Life Science, St. Louis, MO, USA) at 37°C for 1 h. Slides were then washed in decreasing salt solutions (2×, 1× and 0.5× SSC) and a high-stringency wash was performed in 0.1× SSC at 65°C for 1 h. The slides were then cooled to room temperature before being washed twice in Buffer 1 (100 mM Tris, 150 mM NaCl, pH 7.5). The slides were blocked in Buffer 1 containing 1% goat serum and 0.1% Triton X-100 for 1 h. Slides were incubated overnight at room temperature with a sheep anti-digoxigenin antibody conjugated to alkaline phosphatase (Anti-Digoxigenin-AP Fab fragments, Roche Diagnostics, Indianapolis, IN, USA) diluted 1:5000 in fresh blocking buffer. The next day the slides were washed three times in Buffer 1, followed by one wash in alkaline substrate buffer (ASB; 100 mM NaCl, 100 mM Tris, 50 mM MgCl_2_, pH 9.5). Digoxigenin-labeled products were revealed by a color reaction that contained 5% polyvinyl alcohol, 1 mM levamisole, and 2% NBT/BCIP (Roche Diagnostics, Indianapolis, IN, USA) in ASB buffer. Color reactions were stored in the dark until the cells were labeled dark purple. The length of the color reaction was probe specific and varied between 6–8 h. To terminate the reaction, the slides were washed extensively in deionized water and the antibody was stripped with a 10-min incubation in 0.2 M glycine, pH ~2. Slides were then fixed in 2.5% glutaraldehyde in Buffer 1 for 1 h. Slides were washed extensively in deionized water, ethanol dehydrated and then air-dried. For detection of ^35^S-CRH-BP signal, slides were dipped in Ilford K5D nuclear emulsion (Polyscience, Warrington, PA, USA) and stored in the dark for 2–3 weeks at 4°C. Slides were developed in Kodak D19 developer for 3 min, rinsed in water for 30 s, and fixed in Kodak Rapid Fixer for 4 min. Slides were then washed extensively in deionized water, ethanol dehydrated and coverslipped with Permount.

### Data Analysis

The emulsion dipped slides were viewed and analyzed using a Leica DMR microscope (Leica, Wetzlar, Germany). Images were captured using brightfield microscopy (40× objective) and a Zeiss Axiocam 506 color camera. Images were adjusted in Adobe Photoshop (Adobe Systems Inc., San Jose, CA, USA) to increase the detection of signal above background. The cell type-specific expression of CRH-BP in the PFC was quantified at two different Bregma coordinates (Paxinos and Franklin, 2001), 1.94 mm and 2.10 mm relative to Bregma. The following regions of the PFC were quantified: 1.94 mm—cingulate, prelimbic, infralimbic, dorsal peduncular, lateral orbital, agranular insular ventral (AIV), agranular insular dorsal (AID) and dysgranular insular (DI) cortices; 2.10 mm—cingulate, prelimbic, medial orbital, dorsal peduncular, ventral orbital, lateral orbital and agranular insular cortices. To demarcate the different subregions of the PFC, adjacent cresyl violet-stained sections were viewed at 1.6× and regions of interest were outlined according to Paxinos and Franklin (2001) using Stereo Investigator (MBF Bioscience, Williston, VT, USA). This outline was then superimposed on the respective experimental section for analysis. The cell type-specific expression of CRH-BP within each outlined region of the PFC was quantified using a 20× objective and marking positive cells in Stereo Investigator (*n* = 2–3 mice for PV; *n* = 4 mice for all remaining probes). A cell was considered to express CRH-BP mRNA when it contained greater than six silver grains/cell (at least three times above background). A cell was considered digoxigenin-positive when it was clearly labeled purple compared to similarly sized cells in a nearby region (in the same PFC section) that did not express the respective mRNA of interest. For example, deeper cortical layers were used for VIP to define “negative” or background signal as VIP is most highly expressed in the superficial layers of the cortex. Faint purple cells were not counted, which may lead to a small underestimation of colocalization in certain cases of low levels of molecular marker expression. Dual labeled cells were purple with silver grains directly overlaying them. In each region of the PFC, the number of CRH-BP-positive, digoxigenin-positive (PV, SST, VIP and GAD), and dual labeled cells were counted. The percentage of CRH-BP-positive cells that were dual labeled was determined (number of dual labeled cells/the total number of CRH-BP-positive cells × 100). This percentage was averaged between animals for each probe and brain region. For the analysis of CRH-BP mRNA expression in VGLUT or GAD neurons at 1.94 mm, both the right and the left hemispheres were quantified and averaged to generate one value per animal. For the remaining analyses, only one hemisphere (right or left) was quantified, as we did not observe significant differences in colocalization between sides for any probe.

Data were analyzed using a 2-way (brain region × cell type) analysis of variance (ANOVA) and Bonferroni *post hoc* analyses were performed for multiple comparisons when appropriate. All data are reported as means ± SEM and significant values were accepted at *p* < 0.05 for all statistical tests.

## Results

### Colocalization of CRH-BP with VGLUT and GAD

**Figure 1:**
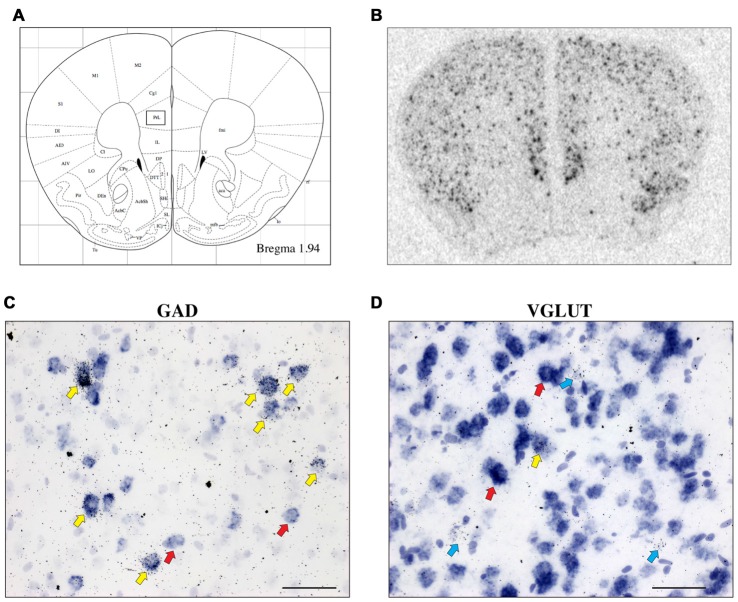
The expression of corticotropin-releasing hormone-binding protein (CRH-BP) mRNA in excitatory (vesicular glutamate transporter (VGLUT)-expressing) and inhibitory (glutamic acid decarboxylase (GAD)-expressing) neurons in the prefrontal cortex (PFC). Coronal section from the Paxinos and Franklin (2001) mouse brain atlas **(A)** at Bregma coordinate 1.94 mm, highlighting the prelimbic region of the PFC where the images in **(C,D)** were captured. An *in situ* autoradiogram **(B)** shows abundant CRH-BP mRNA expression throughout the PFC. Higher magnification bright field images show a high degree of colocalization between CRH-BP and GAD (GAD65 and GAD67) mRNA **(C)** and a low degree of colocalization between CRH-BP and VGLUT (VGLUT1 and VGLUT2) mRNA **(D)** in the prelimbic PFC. Cells labeled for CRH-BP only (silver grains) are indicated with blue arrows, examples of cells labeled for GAD or VGLUT only (purple digoxigenin signal) are indicated with red arrows, and dual-labeled cells are indicated with yellow arrows. Images shown are from prelimbic cortical layers III and V **(C)** and II and III **(D)**. Midline is on the right side of panels **(C,D)**. Scale bar: 50 μm.

**Figure 2:**
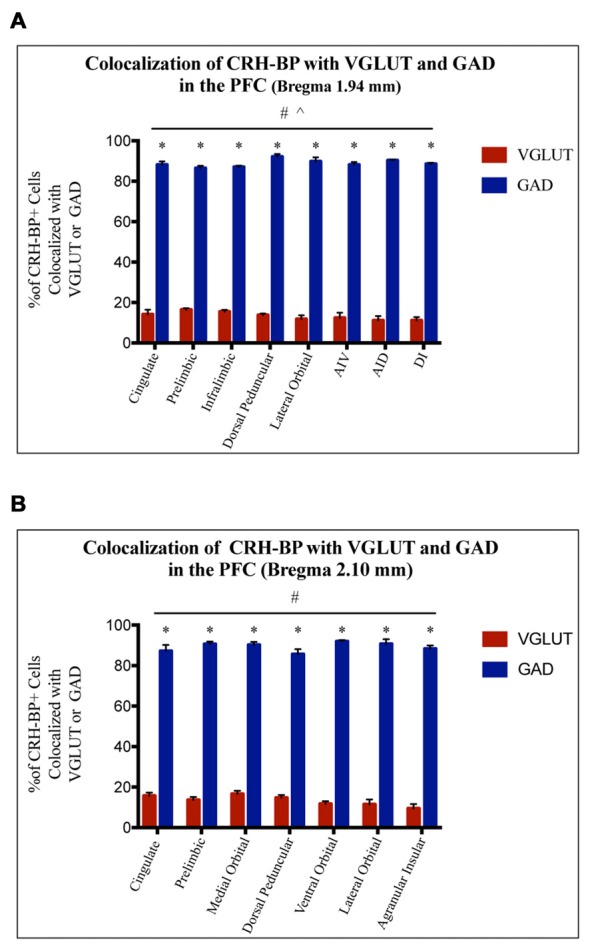
CRH-BP mRNA colocalizes predominantly with GAD in the PFC. Quantification of CRH-BP mRNA colocalization with GAD and VGLUT in the caudal PFC (**A**, Bregma coordinate 1.94 mm) and a more rostral region of the PFC (**B**, Bregma coordinate 2.10 mm). Data represent the percentage of CRH-BP-positive cells colocalized with VGLUT or GAD mRNA. The colocalization between CRH-BP and GAD was significantly higher than the colocalization between CRH-BP and VGLUT at both Bregma coordinates **(A,B)**, as revealed by a significant main effect of cell type (^#^*p* < 0.0001). *Post hoc* analyses showed that the degree of colocalization was significantly higher for GAD compared to VGLUT in all subregions of the PFC at both Bregma coordinates (**A,B**; *p < 0.0001). ^^^*p* < 0.05 interaction. Data represent the mean ± SEM. AIV, agranular insular ventral; AID, agranular insular dorsal; DI, dysgranular insular.

CRH-BP mRNA is readily detected in layers II–VI of the PFC (*in situ* autoradiogram in Figure 1B), consistent with previous studies (Potter et al., 1992; Chan et al., 2000). To determine the cell type-specific expression of CRH-BP in excitatory and/or inhibitory neurons of the PFC, dual *in situ* hybridization was performed to examine the cellular colocalization of CRH-BP mRNA with VGLUT (VGLUT1 and VGLUT2) and GAD (GAD65 and GAD67) mRNA, respectively. VGLUT3 was not included in the analysis, as it is expressed at low levels in the cortex and has been identified in GABAergic interneurons in certain brain regions (Herzog et al., 2004). As expected, VGLUT-expressing cells were more prevalent than GAD-expressing cells in the PFC (Figures 1C,D). For each subregion of the PFC (Figure 1A), the percentage of CRH-BP-positive cells that co-expressed GAD or VGLUT was calculated. In the caudal PFC (Bregma coordinate 1.94 mm), the colocalization between CRH-BP mRNA and GAD mRNA (Figure 1C) was significantly higher than the colocalization between CRH-BP mRNA and VGLUT mRNA (Figure 1D; quantified in Figure 2A), as revealed by a significant main effect of cell type (*F*_(1,48)_ = 9591, *p* < 0.0001). *Post hoc* analyses revealed that CRH-BP mRNA is significantly more colocalized with GAD compared to VGLUT in all subregions of the PFC at this Bregma coordinate (*p* < 0.0001). The percentage of CRH-BP-expressing cells colocalized with GAD mRNA did not vary greatly between PFC subregions (Figure 2A), with the highest percentage of colocalization in the dorsal peduncular cortex (92.1 ± 1.4%) and the lowest percentage in the prelimbic cortex (86.5 ± 1.2%). Likewise, the percentage of CRH-BP-expressing cells colocalized with VGLUT mRNA did not vary greatly between subregions (Figure 2A); the highest degree of colocalization was detected in the prelimbic cortex (16.5 ± 0.8%) compared to the lowest degree of colocalization in the dorsal agranular insular cortex (11.1 ± 2.2%).

At a more rostral portion of the PFC (Bregma coordinate 2.10 mm), there was a similar pattern of colocalization, with a higher degree of colocalization between CRH-BP and GAD compared to VGLUT (Figure 2B), as revealed by a significant main effect of cell type (*F*_(1,42)_ = 5675, *p* < 0.0001). *Post hoc* analyses revealed that CRH-BP mRNA is significantly more colocalized with GAD compared to VGLUT in all subregions of the PFC at this Bregma coordinate (*p* < 0.0001). Similar to PFC at Bregma 1.94 mm, the percentage of CRH-BP-expressing neurons colocalized with GAD did not vary greatly between each PFC subregion (Figure 2B); the highest degree of colocalization was detected in the ventral orbital cortex (92 ± 0.7%) compared to the lowest percentage in the dorsal peduncular cortex (85.6 ± 2.5%). Similarly, the percentage of CRH-BP-expressing cells colocalized with VGLUT mRNA did not vary greatly between PFC subregion (Figure 2B), with the highest degree of colocalization in the medial orbital cortex (16.6 ± 1.6%) and the lowest degree of colocalization in the agranular insular cortex (9.5 ± 2.2%). Finally, the percentage of GAD-positive cells expressing CRH-BP mRNA in the PFC was estimated to be 33% (range: 20%–40%).

### Colocalization of CRH-BP with Interneuron Molecular Markers

**Figure 3:**
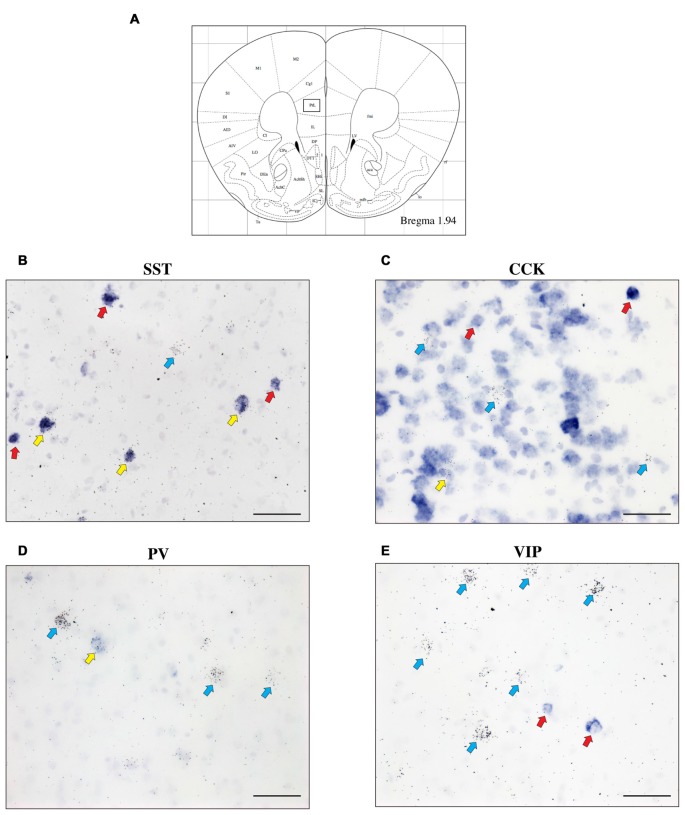
The expression of CRH-BP mRNA in various GABAergic interneuron subtypes in the PFC. Coronal section from the Paxinos and Franklin (2001) mouse brain atlas **(A)** at Bregma coordinate 1.94 mm, highlighting the prelimbic region of the PFC where the images in **(B–E)** were captured. High magnification bright field images show a high degree of colocalization between CRH-BP and somatostatin (SST) mRNA **(B)** and a lower degree of colocalization between CRH-BP and cholecystokinin (CCK) **(C)**, parvalbumin (PV) **(D)** and vasoactive intestinal peptide (VIP) **(E)** in the prelimbic PFC. Cells labeled for CRH-BP only (silver grains) are indicated with blue arrows, examples of cells labeled for SST, CCK, PV, or VIP only (purple digoxigenin signal) are indicated with red arrows, and dual-labeled cells are indicated with yellow arrows. Images shown are from prelimbic cortical layers II—V **(B)**, I—III **(C)**, V and VI **(D)** and I—III **(E)**. Midline is on the ride side of panels **(B–E)**. Scale bar: 50 μm.

**Figure 4:**
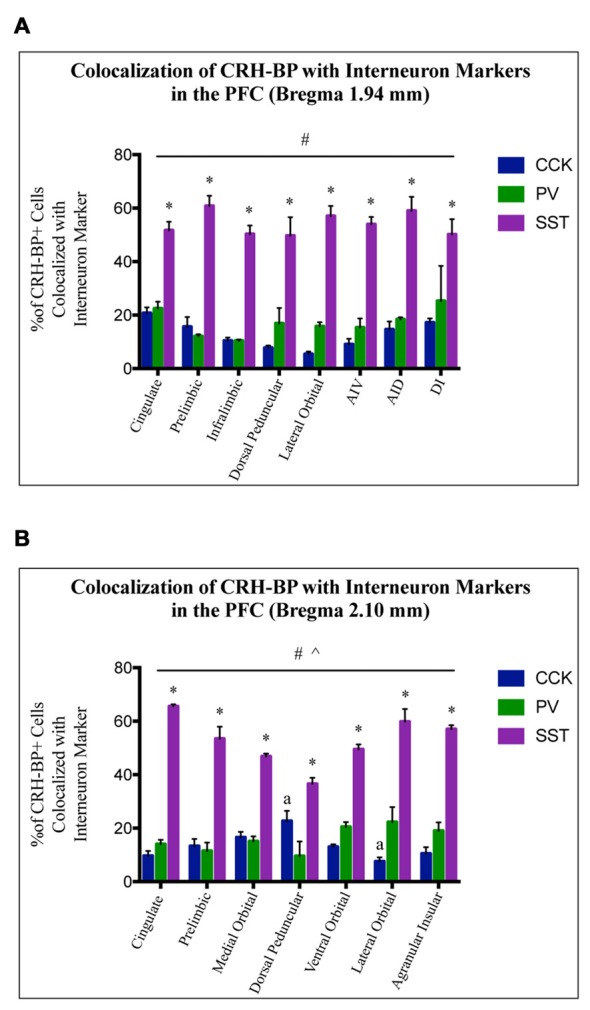
CRH-BP mRNA colocalizes predominantly with SST interneurons in the PFC. Quantification of CRH-BP mRNA colocalization with CCK, PV and SST in the caudal PFC (**A**, Bregma coordinate 1.94 mm) and a more rostral region of the PFC (**B**, Bregma coordinate 2.10 mm). Data represent the percentage of CRH-BP-positive cells colocalized with interneuron marker. Data for the colocalization between CRH-BP and VIP is not shown, as colocalization was barely detectable (see text). There was a significant main effect of cell type (^#^*p* < 0.0001) at both Bregma coordinates **(A,B)**. *Post hoc* analyses showed that the colocalization between CRH-BP and SST was significantly higher than the colocalization of CRH-BP with CCK, PV, or VIP in all subregions of the PFC at both Bregma coordinates (*p < 0.01 compared to CCK, PV and VIP). ^^^*p* < 0.0001 interaction. ^a^*p* < 0.05 compared to respective PV group. Data represent the mean ± SEM. AIV, agranular insular ventral; AID, agranular insular dorsal; DI, dysgranular insular.

To further characterize the cell type-specific expression of CRH-BP in inhibitory neurons of the PFC, dual *in situ* hybridization was performed to examine the cellular colocalization of CRH-BP with molecular markers of GABAergic interneurons, including the calcium-BP PV and the neuropeptides CCK, SST and VIP (Figure 3). These markers represent four largely distinct subclasses of interneurons (partial overlap between CCK and VIP) that account for the majority of interneurons in the frontal cortex (Kawaguchi and Kubota, 1997; Tremblay et al., 2016). CCK-expressing cells were the most abundant in the PFC, followed by SST, PV and VIP. PV-expressing cells were more prevalent in the lateral PFC compared to the medial PFC. For each subregion of the PFC, the percentage of CRH-BP-positive cells that expressed each interneuron marker was calculated (Figure 4). In the caudal PFC (Bregma coordinate 1.94 mm), a 2-way ANOVA revealed a significant main effect of cell type (*F*_(2,56)_ = 300, *p* < 0.0001). *Post hoc* analyses revealed that the colocalization between CRH-BP and SST (Figure 3B) is significantly higher than the colocalization between CRH-BP and CCK (Figure 3C), PV (Figure 3D), or VIP (Figure 3E) in all subregions of the PFC at this Bregma coordinate (*p* < 0.0001; Figure 4A). The percentage of CRH-BP-expressing cells colocalized with SST ranged between 49.7% and 60.8% (Figure 4A). The percentage of CRH-BP-positive cells colocalized with CCK did not significantly differ from the percentage colocalized with PV within each subregion of the PFC (Figure 4A). The range of CRH-BP colocalization in the PFC was 10.4%–25.3% for PV and 5.5%–20.7% for CCK. There were no CRH-BP-positive cells that expressed VIP in the PFC at this Bregma coordinate (0% colocalization), therefore VIP was not included in Figure 4A or the statistical analyses. All images in Figure 3 are from prelimbic PFC (Bregma 1.94 mm, layers indicated) and the distribution of markers in the prelimbic PFC at this Bregma coordinate is largely consistent with previous reports (Pinto and Dan, 2015; Whissell et al., 2015; Ueno et al., 2017) and Allen Mouse Brain Atlas (mouse.brain-map.org). It should be noted that both low and high power magnification images were examined for each marker across the PFC (from both single and dual *in situ* hybridization experiments) to confirm the specificity and sensitivity of each molecular marker probe. In addition, radioactive *in situ* hybridization experiments were also performed for VIP, SST, PV and CCK in PFC with similar results to the digoxigenin-labeled molecular marker probes (unpublished data).

At the more rostral portion of the PFC (Bregma coordinate 2.10 mm), a 2-way ANOVA also revealed a significant main effect of cell type (*F*_(2,47)_ = 440, *p* < 0.0001). *Post hoc* analyses showed that the colocalization between CRH-BP and SST is significantly higher than the colocalization between CRH-BP and VIP, CCK, or PV in all subregions of the PFC at this Bregma coordinate (*p* < 0.01; Figure 4B). The percentage of CRH-BP-expressing cells colocalized with SST ranged between 36.6% and 65.6% (Figure 4B). The percentage of CRH-BP-positive cells colocalized with CCK did not significantly differ from the percentage colocalized with PV, except in the dorsal peduncular cortex, where the percentage of CRH-BP colocalization with CCK was significantly higher than PV (*p* < 0.05), and the lateral orbital cortex, where the percentage of CRH-BP colocalization with PV was significantly higher than CCK (*p* < 0.001; Figure 4B). The range of CRH-BP colocalization in the PFC was 9.6%–22.3% for PV and 7.6%–22.7% for CCK. Only 1 CRH-BP-positive cell was detected that coexpressed VIP in the PFC at this Bregma coordinate, located in the agranular insular cortex (0.26% colocalization; not included in Figure 4B or the statistical analyses).

The percentage of SST- and PV-positive cells that expressed CRH-BP mRNA was also calculated for each subregion of the PFC. In the caudal PFC (Bregma coordinate 1.94 mm), SST colocalization with CRH-BP was 48.1% (range: 38.9%–56.3%) and PV colocalization with CRH-BP was 48.6% (range: 33.5%–64.6%; data not shown). At the more rostral portion of the PFC (Bregma coordinate 2.10 mm), SST colocalization with CRH-BP was 54.1% (range: 34.1%–81% and PV colocalization with CRH-BP was 40.3% (range: 18.4%–87.5%; data not shown).

## Discussion

In the current study, we characterized the expression of CRH-BP in excitatory and inhibitory neurons of the PFC. First, we examined the cellular colocalization of CRH-BP mRNA with GAD (GAD65/GAD67) and/or VGLUT (VGLUT1/VGLUT2) in different regions of the PFC. We found that CRH-BP is highly colocalized with GAD in all regions of the PFC at both Bregma coordinates. To further characterize the expression of CRH-BP in inhibitory interneurons, we examined the colocalization of CRH-BP with various interneuron molecular markers, including SST, CCK, PV and VIP. CRH-BP was colocalized predominantly with SST in all regions of the PFC, while the degree of colocalization was lower for PV and CCK, and barely detectable for VIP. To our knowledge, this study represents the first anatomical characterization of CRH-BP expression in multiple GABAergic interneuron cell types in the rodent PFC.

The PFC contains both glutamatergic pyramidal neurons and GABAergic inhibitory neurons. GABAergic interneurons project locally within the PFC and function to regulate the output of cortical pyramidal neurons. In the present study, we found that CRH-BP mRNA is highly colocalized with GAD, revealing the presence of CRH-BP in GABAergic interneurons of the PFC. Previous characterization of CRH-BP expression in inhibitory neurons of the rodent brain has focused on the VTA. Wang and Morales (2008) demonstrated by dual *in situ* hybridization that CRH-BP mRNA is present in both GABAergic interneurons and dopaminergic neurons of the VTA. The authors found that 27% of CRH-BP-expressing cells co-expressed GAD, and 28% of GAD-expressing cells colocalized with CRH-BP. The presence of CRH-BP in inhibitory interneurons suggests that CRH-BP may act locally within the PFC and VTA to produce some of its effects. Future studies should investigate the expression of CRH-BP in excitatory and inhibitory neurons in other regions of the stress and reward system.

The specific interneuron subtype in which CRH-BP is expressed has important implications for its role within circuits of the PFC, as each interneuron subtype exhibits unique morphology, physiology and connectivity, allowing for precise spatiotemporal control over pyramidal cell activity (reviewed in Tremblay et al., 2016). For example, PV-expressing neurons consist of fast-spiking basket cells or chandelier cells that tend to innervate the soma and proximal dendrites or the axon initial segment of pyramidal neurons, respectively, resulting in strong inhibition (Kawaguchi and Kubota, 1997). SST neurons are adapting regular-spiking or burst spiking Martinotti cells that innervate the distal dendrites of pyramidal cells in layer 1 of the cortex (Kawaguchi and Kubota, 1997). There are also non-Martinotti SST-expressing cells that innervate pyramidal neurons or PV interneurons in other layers of the cortex (Ma et al., 2006; Xu et al., 2013). Based on the strong excitatory input they receive, SST neurons tend to exhibit activity-dependent inhibition of pyramidal neurons (Silberberg and Markram, 2007). VIP neurons are often bipolar and have a variety of firing patterns, including irregular spiking, bursting and rapidly adapting (Kawaguchi and Kubota, 1997). Interestingly, VIP neurons tend to preferentially target SST neurons in the cortex, which may result in disinhibition and increased pyramidal cell activity (Pi et al., 2013). Lastly, CCK neurons are regular or burst spiking basket cells that target the soma and proximal dendrites of pyramidal cells (Kawaguchi and Kubota, 1997).

In the current study, we examined the cellular colocalization of CRH-BP with SST, PV, CCK and VIP in the PFC. SST, PV and VIP represent non-overlapping classes of interneurons in the cortex (Kawaguchi and Kubota, 1997; Xu et al., 2010). CCK and VIP belong to a larger class of interneurons, those that express the ionotropic serotonin receptor 5HT3aR. These interneurons are distinct from the PV and SST interneuron classes, but CCK and VIP interneurons do show some overlap; small CCK basket cells are VIP-positive and large CCK basket cells are VIP-negative (Kawaguchi and Kubota, 1997; Lee et al., 2010). The 5HT3aR class of interneurons includes neurons that express other markers, such as calretinin and reelin (Lee et al., 2010), which were not included in our analyses. In the present study, we determined that CRH-BP is largely expressed in SST interneurons in all regions of the PFC, with a lower, but significant, level of coexpression in PV and CCK interneurons. Given that we observed essentially no colocalization between CRH-BP and VIP in the PFC, it is likely that CRH-BP is expressed in CCK-expressing neurons that do not express VIP.

The observation that CRH-BP is localized in SST neurons is consistent with a recent study by Li et al. (2016). In this study, the authors utilized oxytocin receptor-Cre mice crossed to EGFP-L10a mice and translational ribosome affinity purification profiling. They observed that CRH-BP is highly enriched in oxytocin receptor interneurons of the mPFC. Interestingly, these neurons were identified as a specific subpopulation of SST interneurons (Nakajima et al., 2014; Li et al., 2016), demonstrating the presence of both CRH-BP, SST and oxytocin receptors within the same interneurons in the mPFC. The authors further show that in the male mPFC, CRH enhances the activity of layer II/III pyramidal neurons, an effect that is blocked by application of CRH-R1 antagonist (Li et al., 2016), consistent with previous studies that have demonstrated CRH-mediated enhancement of cortical pyramidal cell activity through CRH-R1 (Gallopin et al., 2006). Strikingly, inhibition of CRH-BP with CRH_6–33_ (a peptide that does not activate CRH receptors), both alone and in the presence of CRH, significantly increased pyramidal cell activity in the male mPFC (Li et al., 2016), suggesting that CRH-BP normally inhibits CRH-R1 activation on layer II/III pyramidal neurons. These data, and our observation of high CRH-BP and SST colocalization, suggest that CRH-BP may be released from SST/oxytocin receptor interneurons and bind CRH to inhibit CRH-R1 activation on prefrontal pyramidal neurons, thus regulating their activity. CRH-BP modulation of CRH-R1 activity on pyramidal neurons may mediate anxiety-like behavior, as conditional knockdown of CRH-BP in oxytocin receptor interneurons in the mPFC increased anxiety in male (but not female) mice (Li et al., 2016). Similarly, selective deletion of CRH-R1 in forebrain glutamatergic neurons reduces anxiety-like behavior (Refojo et al., 2011). As noted above, CRH is expressed in SST-, VIP-, and CCK-positive interneurons (Gallopin et al., 2006; Kubota et al., 2011; Mardinly et al., 2016). Thus, CRH-BP and CRH may be released from the same SST interneurons or, more likely, from different classes of SST, CCK, and/or VIP interneurons. CRH from extracortical sources is likely also acting within the PFC. Future studies should investigate the colocalization between CRH-BP, CRH, and various interneuron subtypes in the mouse PFC to begin to address these questions.

In the current study, we also find a low-to-moderate degree of colocalization between CRH-BP and PV (about 10%–25% of CRH-BP-expressing cells colocalize with PV and ~33%–65% of PV-positive cells colocalize with CRH-BP in caudal PFC in mice). In a recent study, Lake et al. (2016) sequenced RNA molecules from individual neuronal nuclei of a human post-mortem brain to begin to define neuronal subtypes in the human cortex. In their data set, they discovered an interneuron subtype that expresses both PV and CRH-BP, consistent with our observation of CRH-BP mRNA in PV-expressing cells. However, they did not observe CRH-BP and SST co-expression in their samples (Lake et al., 2016). This difference may reflect species differences or perhaps region-specific differences, as the sequencing data originated from six different regions of the cerebral cortex (Lake et al., 2016). It should be noted that PV expression is enriched in areas of the cortex that were not quantitated in our study, including the motor and sensory cortices (Whissell et al., 2015). We also observed greater PV expression in the lateral PFC compared to the mPFC, which may have contributed to the greater colocalization observed between CRH-BP and PV in the lateral PFC compared to the mPFC. Finally, PV expression in our dual *in situ* hybridization experiments was slightly weaker than previous preliminary experiments. Therefore, we may be slightly underestimating the colocalization between CRH-BP and PV in the mouse PFC.

In summary, the current results expand our knowledge on the molecular phenotype of CRH-BP neurons and begin to define the prefrontal cortical circuits in which it’s expressed. We found that CRH-BP is expressed predominantly in GABAergic interneurons of the PFC, particularly SST-expressing interneurons. Based on the findings from this study and previous studies (Gallopin et al., 2006; Refojo et al., 2011; Li et al., 2016), we postulate that CRH-BP may be released from SST interneurons to regulate the activity of pyramidal neurons via CRH-R1, potentially influencing anxiety-like behavior. SST interneurons are sensitive to stress and have been implicated in a variety of stress-related psychiatric disorders, including anxiety and depression (Stengel et al., 2013; Soumier and Sibille, 2014; Lin and Sibille, 2015; Fuchs et al., 2017). Interestingly, CRH-BP appears to be similarly coexpressed in SST neurons in both prelimbic and infralimbic regions of the PFC, areas that have been shown to exhibit opposing effects on stress-related behaviors. The current characterization of the cell type-specific expression of CRH-BP in the PFC will serve as the basis for future studies to manipulate CRH-BP in a cell type- and subregion-specific manner to begin to define its role(s) in psychiatric disorders.

## Author Contributions

KDK and AFS designed the study and wrote the article. KDK and NSH performed the cloning reactions and dual *in situ* hybridization experiments. KDK performed the data analysis.

## Conflict of Interest Statement

The authors declare that the research was conducted in the absence of any commercial or financial relationships that could be construed as a potential conflict of interest.
